# Environmental DNA for biomonitoring

**DOI:** 10.1111/mec.16023

**Published:** 2021-06-27

**Authors:** Jan Pawlowski, Aurélie Bonin, Frédéric Boyer, Tristan Cordier, Pierre Taberlet

**Affiliations:** ^1^ Department of Genetics and Evolution University of Geneva Geneva Switzerland; ^2^ Institute of Oceanology Polish Academy of Sciences Sopot Poland; ^3^ ID‐Gene Ecodiagnostics Geneva Switzerland; ^4^ Department of Environmental Science and Policy Università degli Studi di Milano Milan Italy; ^5^ Laboratoire d'Ecologie Alpine (LECA) CNRS Université Grenoble Alpes Grenoble France; ^6^ NORCE Climate NORCE Norwegian Research Centre AS Bjerknes Centre for Climate Research Bergen Norway; ^7^ Tromsø Museum UiT – The Arctic University of Norway Tromsø Norway

In 2012, Molecular Ecology published a special issue on environmental DNA, which provided an overview of the field of eDNA research and presented a selection of papers on eDNA studies (Taberlet et al., 2012). This special issue also introduced the concept of Biomonitoring 2.0, advocating for the use of DNA‐based identification of taxa in biodiversity surveys and ecosystem assessment (Baird & Hajibabaei, 2012). Since then, hundreds of papers have been published covering various aspects of eDNA‐based biomonitoring from single‐species detection to community studies and environmental impact assessments. Numerous reviews have summarized these studies for both freshwater and marine environments (Bohmann et al., 2014; Thomsen & Willerslev, 2015).

The progress made in the eDNA field during these last ten years has been spectacular (Taberlet et al., 2018). Although the basic concepts and workflow of DNA barcoding and metabarcoding have not changed, the technological advances in high‐throughput sequencing have greatly facilitated the access to eDNA data. It has become possible to monitor biodiversity with unprecedented precision and depth. Massive environmental genomic datasets have been rapidly generated at relatively low cost. The analysis of these datasets using machine learning and other taxonomy‐free approaches opened wide the doors for using new groups of bioindicators to infer ecological status (Cordier et al., 2018, 2019; Pawlowski et al. 2018). At the same time, constant efforts to fill gaps in barcoding reference databases considerably increased the effectiveness of taxonomic identification of eDNA data (Weigand et al., 2019).

Astonishingly, these rapid advances in eDNA‐based technologies are rather timidly implemented in routine biomonitoring (Hering et al., 2018; Shackleton et al., 2021). Although the concept of Biomonitoring 2.0 is widely endorsed, its acceptance in practice is hampered for various reasons. There is no consensus whether eDNA‐based biomonitoring should only apply to conventional bioindicators (Renovate) or should also include new bioindicators (Rebuild) or new taxonomy‐free approaches (Revolutionize) (see Figure 1). Moreover, three main steps on the roadmap from eDNA to biomonitoring are not developed equally. The main attention is given to the development and optimization of eDNA data generation and analysis. The standardization of eDNA methods and their translation into legislatory framework remain at a very early stage. One of the main issues impeding the application of eDNA‐based tools concerns the lack of congruence between the results of traditional and molecular analyses (Aylagas et al., 2020). It is expected that the new method is “safe to use” only if it provides the same or almost same results as the conventional one. However, obtaining such perfect congruence is often impossible because the character of data is very different (e.g., abundance of individuals vs abundance of eDNA reads). Moreover, the eDNA “ecology” can hardly be translated directly into species ecology. There are also numerous biological and technical biases that can affect the generation and processing of eDNA data, impacting their interpretation.

**FIGURE 1:**
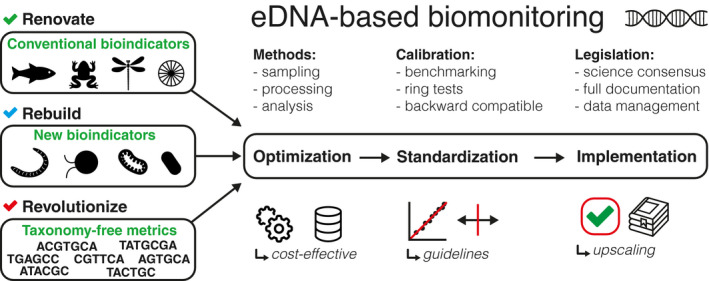
Framework for an eDNA‐based biomonitoring. The boxes on the left represent the main current research avenues, that are mostly focused on particular components of biological communities. The right side represents the milestones to meet for the implementation of eDNA for routine biomonitoring

This special issue addresses some of these challenges by presenting the latest advances in eDNA field and discussing their strengths and limitations when applied to routine biomonitoring. The issue comprises 29 papers grouped into four sections and covering different aspects of eDNA applications. It is accompanied by an opinion paper, which clarifies the eDNA terminology in relation to its use in biomonitoring (Pawlowski et al., 2020). The first section comprises a series of studies using new analytical tools (e.g. machine learning), new types of bioindicators and genomic data (e.g. shotgun sequencing) for the assessment of ecological status. It is followed by a section dedicated to fish eDNA, whose application in biomonitoring is the most advanced. The third section comprises papers dealing with various methodological aspects and the comparison between conventional and molecular methods. The final section presents few examples of eDNA applications for biodiversity surveys and population genetics.

## 1 NOVEL APPROACHES TO MONITOR ECOSYSTEMS

The development of environmental genomics enables monitoring of microbial and meiofaunal communities that were previously inaccessible when using conventional methods. However, our knowledge of the ecology of these communities is very limited and therefore new analytic approaches are necessary to integrate them into routine bioassessment. This section begins with a review of implementation strategies for the application of environmental genomics in ecological diagnostics (Cordier et al., 2021). The authors introduce four broad categories of possible strategies, including (1) DNA‐based taxonomic identification of known bioindicators, (2) taxonomy‐free discovery of new bioindicators, (3) structural community metrics, and (4) functional community metrics. Each of these strategies is adapted to a particular type of data (metabarcoding, metagenomics, metatranscriptomics) and rely on different computational analyses in order to provide an assessment of the ecological status.

Among the different analytical tools, machine learning seems to be the most promising way to predict the ecological status (Cordier et al., 2018, 2019). In this issue, its performance is tested in the case of the benthic diatoms index widely used in the assessment of ecological quality of rivers and streams (Apothéloz‐Perret‐Gentil et al., 2021). This study shows that supervised machine learning performs better than the taxonomic assignment, but its predictions are similar to those obtained using a taxonomy‐free molecular assignment approach. Moreover, the efficiency of a taxonomic assignment method strongly depends on the completeness of the reference database, highlighting the need to fill in the existing gaps, particularly in the case of bioindicator taxa.

The ability of *de novo* prokaryotic bioindicators to predict multiple anthropogenic impacts on estuarine and coastal benthic communities is demonstrated by Lanzén et al. (2021). The authors compare their results to the traditional macrofauna‐based indices and discuss various advantages of using microbial bioindicators as they are more sensitive to different abiotic pressures. Similar conclusions were reached in the case of environmental impact assessment of marine aquaculture (Frühe et al., 2021) and the oil and gas industry (Mauffrey et al., 2021). Both studies demonstrate the effectiveness of machine learning and *de novo* microbial bioindicators and promote their use for benthic monitoring in marine environments.

The last two papers in this series explore new directions for the further development of ecogenomic diagnostics. Broman et al. (2021) use environmental RNA (eRNA) shotgun sequencing to analyse the impact of organic enrichment on benthic micro‐eukaryotic communities. Compared to eDNA metabarcoding that is used in the majority of studies, eRNA shotgun data has the advantage to overcome the potential biases of PCR amplification and to better capture the organismic response to environmental pressures by targeting predominantly active cells. Ibrahim et al. (2021) use historical eDNA metabarcoding data to analyze the impact of eutrophication on lake phytoplankton in the 20th century. This study demonstrates the potential of paleo‐metabarcoding to characterize past biodiversity and establish reference conditions for future monitoring.

## 2 REFINING FISH eDNA SURVEYS

The second series of papers concerns the use of eDNA to monitor fish diversity. We focus on fish because they are among the most important groups of bioindicators and also because their study from an eDNA perspective is the most advanced (Pont et al., 2021). The barcoding reference database of common fish species in some regions is close to completeness (Knebelsberger et al., 2015), fish‐specific markers are well defined (M. Miya et al., 2015; Valentini et al., 2016; Zhang et al., 2020) and protocols for fish eDNA sampling and processing are well established (Masaki Miya et al., 2020; Valentini et al., 2016). Currently, considerable efforts are directed to solve the most challenging issue, which is related to quantitative fish eDNA data and its application for inferring fish indices in routine biomonitoring.

Two papers address this issue by proposing different approaches to estimate fish abundance from eDNA data. Fukaya et al. (2021) use numerical hydrodynamic models to simulate the spatial and temporal distribution of fish eDNA in aquatic environments. By integrating the models to the measures of eDNA concentration, the authors obtained estimates of fish population abundance comparable to those obtained by the quantitative echo sounder method. Yates et al. (2021) improve the correlation between eDNA concentration and fish abundance by integrating allometric scaling coefficients. Such coefficients can help adjust the values of eDNA production taking in consideration density, biomass and metabolic rates characteristic to a given taxon.

A better understanding of the “ecology” of fish eDNA, and particularly how its temporal and spatial distribution is shaped by abiotic and biotic factors, is the subject of the following papers. Littlefair et al. (2021) tested how seasonal variations in thermal stratification influence the distribution of fish eDNA in lakes. The authors show that eDNA distribution follows lake stratification and the thermal niche of the species, which in turn may affect its detection in certain seasons. The distribution of fish and amphibian eDNA in a lentic system was investigated experimentally by Brys et al. (2021). This study indicates high eDNA decay rates and limited dispersal, reinforcing the accuracy of eDNA‐based monitoring for retrieving the spatiotemporal occupancy patterns. The advantages of using eDNA for survey of fish populations were also demonstrated by other papers in this section. McColl‐Gausden et al. (2021) showed that eDNA metabarcoding is generally more sensitive than electrofishing for conducting fish surveys in freshwater streams, while Aglieri et al. (2021) demonstrate strong complementarity of eDNA‐based analysis with visual and capture‐based methods in the survey of coastal fish communities.

## 3 METHODOLOGY AND COMPARISON WITH CONVENTIONAL METHODS

General acceptance of molecular methods in biomonitoring requires their benchmarking against conventional morphotaxonomy‐based approaches. This is commonly achieved by processing the same samples in parallel using different methods and by assessing how the molecular data fit to the results of traditional approaches, considered as a ground truth. The papers of this section compare the results of eDNA metabarcoding vs bulk DNA metabarcoding vs different morphology‐based approaches. They also present and discuss the biases of molecular methods and propose solutions to improve the outcomes of molecular data generation and processing.

The section begins with the three comparative studies of marine biomonitoring. Suter et al. (2021) evaluate the performance of water eDNA and bulk DNA metabarcoding in assessing the biodiversity of zooplankton in open ocean, currently monitored by using continuous plankton recorders. The study shows that both methods recover more species than morphological analyses, however, their efficiency depends on the sampling method and selected marker. They conclude that eDNA metabarcoding is very promising, but it still requires some refinement and standardization before it can be routinely used for zooplankton biomonitoring. Similar conclusions are drawn from the comparison of sediment DNA metabarcoding and macrofauna surveys applied to monitor benthic impacts of salmon farms (He et al., 2021). Although the authors found a certain coherence in relative abundance of common macrofauna bioindicators inferred from morphological and eDNA data, they observed that the correlation with organic enrichment was much stronger for meiofauna, which is not usually included in biomonitoring studies. Significant differences were also found between water eDNA samples and bulk DNA extracts from adjacent benthic communities (Antich et al., 2021). The authors concluded that water eDNA is a poor proxy for the analysis of benthic communities, although they do not exclude that the use of taxon‐specific markers could improve the congruence between eDNA and bulk DNA metabarcoding data.

The importance of marker selection has also been emphasized in the case of freshwater macrobenthos metabarcoding. The performance of different markers, with focus on key insect orders (Ephemeroptera, Plecoptera and Trichoptera) was tested by Ficetola et al. (2021). The authors demonstrate the complexity of the marker selection process and advocate for the use of multiple markers to cover the widest range of taxa. Combining data from different markers was shown to considerably improve the match between macrobenthic indices inferred from bulk DNA and morphotaxonomic surveys (Meyer et al., 2021). A multimarker approach was also recommended for the assessment of macroinvertebrate communities from the bulk preservative (Martins et al., 2021). Despite the importance of using multiple markers, the authors also demonstrate that the presence of heavily sclerotized exoskeleton can act as a limiting factor for the detection of some taxa.

The comparison of bulk DNA vs water eDNA metabarcoding has been reported by two papers. Gleason et al. (2021) show that bulk DNA metabarcoding more accurately represents the local stream macroinvertebrate community, with water eDNA data being overwhelmed by non‐metazoan sequences. The same difference was observed when comparing bulk DNA to water eDNA and morphological inventories of pond macroinvertebrates (Harper et al., 2021). However, the authors consider both approaches as complementary and suggest that they should be combined for comprehensive assessment of the invertebrate community. The importance of bulk DNA metabarcoding as a tool for the assessment of marine ecosystems is also highlighted by van de Loos and Nijland (2021). The authors review various technical biases affecting bulk DNA metabarcoding workflow and discuss possible improvements that could help overcoming these biases in the future.

The analysis of water samples from five sites in the Brazilian Atlantic forest and one adjacent site in Cerrado grasslands allowed Lopes et al. (2020) to demonstrate that eDNA metabarcoding significantly improves traditional monitoring methods, confirming the presence of frog species undetected by traditional methods. For a few years, invertebrate‐derived DNA (iDNA) from leech blood‐meal have been used to track mammalian species (Schnell et al., 2012). Here, Drinkwater et al. (2021) apply this approach to assess differences in mammalian diversity across a gradient of forest degradation in Borneo. For monitoring elusive mammals, the iDNA method complements the more traditional and widely used camera trapping.

The last two papers in this section provide examples of metabarcoding optimizations aiming at improving its effectiveness in biomonitoring surveys. Guerrieri et al. (2021) show how soil preservation methods can affect estimates of taxonomic richness and community composition. The authors propose guidelines for optimizing soil preservation conditions in agreement with the objectives and practical constraints of the research project. On the other hand, Mächler et al. (2021) address the optimization of data analysis, by investigating how stringency filtering can affect eDNA diversity estimates. The authors conclude that the use of Hill numbers can help in comparisons of eDNA datasets that strongly differ in diversity.

## 4 Other perspectives for eDNA‐based biomonitoring

The last three articles in this special issue present ground‐breaking approaches to monitoring biodiversity. Martel et al. (2021) clearly show that eDNA surveys paired with occupancy modelling can uncover metapopulation dynamics and their drivers. Such type of information is important for monitoring endangered species distributed in metapopulations and is quite difficult to obtain via traditional inventories. Shum and Palumbi (2021) reanalyzed a published marine metabarcoding dataset concerning cobble communities found within kelp forest ecosystems. They focussed on diversity data at the intraspecific level to infer population structure and demographic trends. This type of approach greatly increases the scope and value of metabarcoding studies, also opening the way towards metaphylogeography (Turon et al., 2020). Finally, Sigsgaard et al. (2021) successfully tracked insects from cow dungs from different environments, and showed that eDNA metabarcoding represents an efficient method for assessing insect diversity, with potential for biomonitoring in relation with the relatively easy standardization of such an approach.

## 5 CONCLUSION

As shown by the collection of papers published in this issue, potential applications of eDNA in biomonitoring are highly diverse. Their scope ranges from tracking endangered species to surveying biodiversity or assessing environmental impact. Some papers focus on integrating eDNA into existing bioindication systems, whereas others use eDNA to expand the range of bioindicators and include inconspicuous, commonly overlooked microbial and meiofaunal taxa. All these papers attest to major efforts that have been done to improve eDNA methodology at every step of the workflow from sampling to data analysis. They also contribute to better understand the biological and technical factors impacting the eDNA analyses. Yet, despite this huge new knowledge and numerous practical advantages, the implementation of eDNA in routine biomonitoring still has not taken off.

It is now high time to move on and to transform the eDNA field into a truly applied science. The biodiversity crisis and global environmental changes call for an urgent modernization of the tools to monitor biodiversity and assess the ecological status of our environment. As shown by the papers published here, the eDNA methodology achieved top levels of technical and scientific excellence in many areas. Certainly, there are some biases and limitations inherent to eDNA specificity, but there is no reason to consider that the technology is less “safe to use” than the conventional morpho‐taxonomic approaches. There are also actions to be taken to ensure the quality and to build confidence in eDNA analyses through standardization of technical protocols and intercalibration tests. However, in view of the substantial efforts that have been made by the scientific community and illustrated by the content of this special issue, it is reasonable to expect that the implementation of eDNA‐based tools in biomonitoring will not be long in coming.

## CONFLICT OF INTEREST

There is no conflict of interest regarding the authors of this paper.
